# PGAP-X: extension on pan-genome analysis pipeline

**DOI:** 10.1186/s12864-017-4337-7

**Published:** 2018-01-19

**Authors:** Yongbing Zhao, Chen Sun, Dongyu Zhao, Yadong Zhang, Yang You, Xinmiao Jia, Junhui Yang, Lingping Wang, Jinyue Wang, Haohuan Fu, Yu Kang, Fei Chen, Jun Yu, Jiayan Wu, Jingfa Xiao

**Affiliations:** 1 CAS Key Laboratory of Genome Sciences and Information, Beijing Institute of Genomics, Chinese Academy of Sciences, Beijing, 100101 People’s Republic of China; 2 Big Data Center, Beijing Institute of Genomics, Chinese Academy of Sciences, Beijing, 100101 People’s Republic of China; 3 University of Chinese Academy of Sciences, Beijing, 100049 People’s Republic of China; 4 Department of Computer Science and Technology, Tsinghua University, Beijing, 100084 People’s Republic of China; 5 Beijing Institute of Genomics, Chinese Academy of Sciences, NO. 1 Beichen West Road, Chaoyang District, Beijing, 100101 People’s Republic of China

**Keywords:** Pan-genomics, Genome visualization, Genetic variation

## Abstract

**Background:**

Since PGAP (pan-genome analysis pipeline) was published in 2012, it has been widely employed in bacterial genomics research. Though PGAP has integrated several modules for pan-genomics analysis, how to properly and effectively interpret and visualize the results data is still a challenge.

**Result:**

To well present bacterial genomic characteristics, a novel cross-platform software was developed, named PGAP-X. Four kinds of data analysis modules were developed and integrated: whole genome sequences alignment, orthologous genes clustering, pan-genome profile analysis, and genetic variants analysis. The results from these analyses can be directly visualized in PGAP-X. The modules for data visualization in PGAP-X include: comparison of genome structure, gene distribution by conservation, pan-genome profile curve and variation on genic and genomic region. Meanwhile, result data produced by other programs with similar function can be imported to be further analyzed and visualized in PGAP-X. To test the performance of PGAP-X, we comprehensively analyzed 14 *Streptococcus pneumonia* strains and 14 *Chlamydia trachomatis*. The results show that, *S. pneumonia* strains have higher diversity on genome structure and gene contents than *C. trachomatis* strains. In addition, *S. pneumonia* strains might have suffered many evolutionary events, such genomic rearrangements, frequent horizontal gene transfer, homologous recombination, and other evolutionary process.

**Conclusion:**

Briefly, PGAP-X directly presents the characteristics of bacterial genomic diversity with different visualization methods, which could help us to intuitively understand dynamics and evolution in bacterial genomes. The source code and the pre-complied executable programs are freely available from http://pgapx.ybzhao.com.

**Electronic supplementary material:**

The online version of this article (doi: 10.1186/s12864-017-4337-7) contains supplementary material, which is available to authorized users.

## Background

Since the pan-genome concept was proposed in 2005 [1, 2], it has been rapidly employed to investigate the bacterial genomic evolution and dynamics in the past decade [3–6]. In these years, it also was widely utilized to perform comparative genomic analysis in virus [7], fungi [8], and plant [9]. To make pan-genome analysis for bacterial genomes more easy and efficient, several programs and databases were developed, including Panseq [10], PGAT [11], PanCGHweb [12], PanGP [13], ITEP [14], PGAP [15], and so on. Early programs or databases mainly focus on limited functional analysis, while PGAP integrates five common analytical modules, including cluster analysis of functional genes, pan-genome profile analysis, genetic variation analysis of functional genes, species evolution analysis and function enrichment analysis of gene clusters. After being released publicly, PGAP has been downloaded for more than 4000 times from more than 60 countries, and it was widely used to perform pan-genome analysis for different bacteria, such as *Mycobacterium* [16], *Bifidobacterium* [17], *Lactococcus* [18] and so on. To our best knowledge, PGAP, together with Panseq, have been reported as the most popular packages at the end of 2014 [19].

However, a never-ending improvement of pan-genomic tools is data interpretation and visualization, which would provide better data mining results and quality graphics for research and publication. In the past years, several standalone programs and web-based servers have been developed to visualize data from pan-genome sight. However, these programs and servers provided very limited functions. Moreover, they cannot present orthologous relationship and genetic variation inside both genomes and genes from genomic structure sight. To address this question, we developed a genome-oriented software, PGAP-X, which will perform pan-genome analysis from genome structure sight. PGAP-X does not only perform data analysis independently, but also directly visualize and interpret result data. Results data generated by other programs with similar function could also be imported to PGAP-X for further analysis and visualization, after being converted to compatible data format. PGAP-X can be used to well analyze and present the diversity of genome structure and gene content for those strains from the same specie or closely related species, which have high similarity in genome structure.

## Methods

### Overview of PGAP-X architecture

**Fig. 1:**
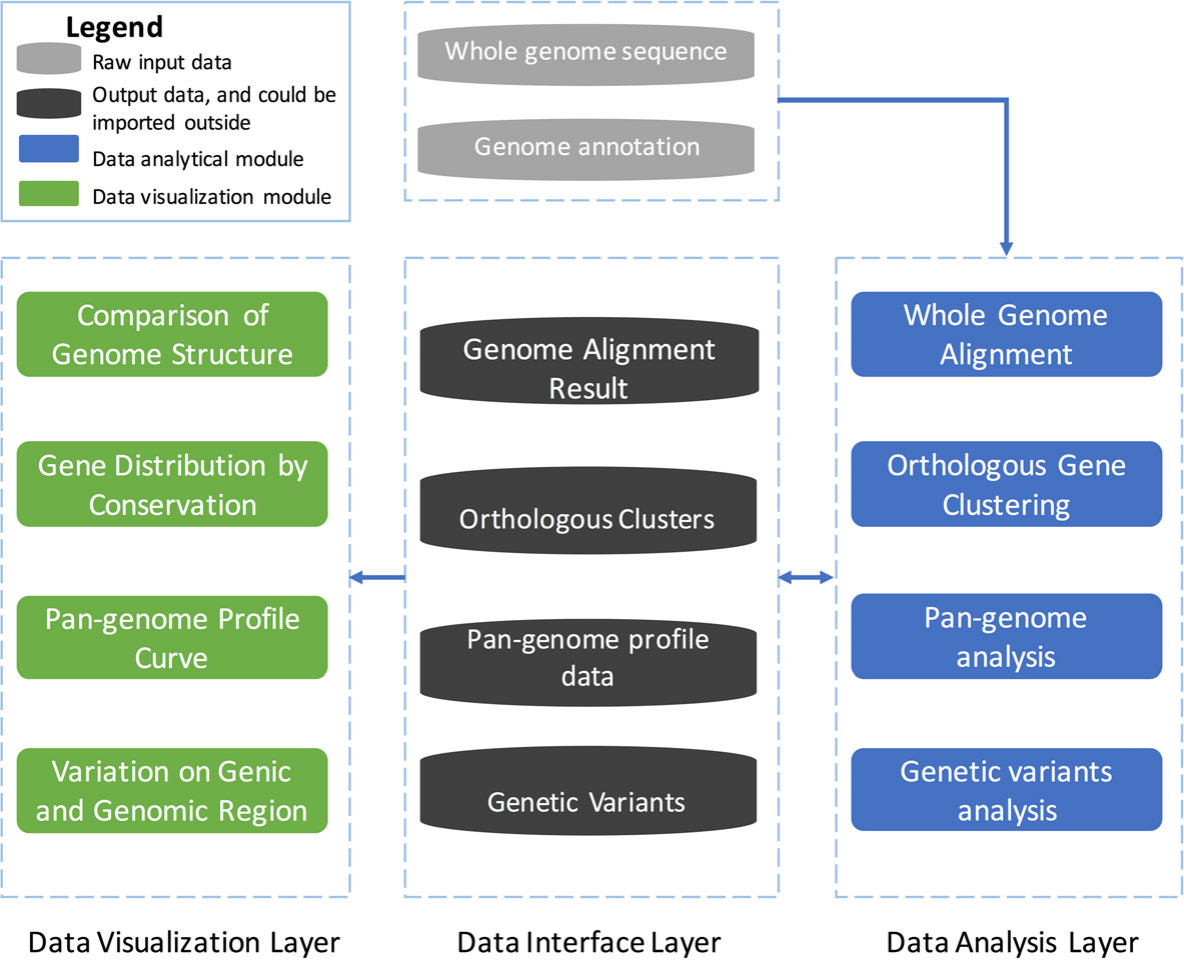
The frame for PGAP-X. All analytical modules are grouped in to three layers: data visualization layer, data interface layer and data analysis layer, which are separated with dashed line. Data interface layer is divided into two parts, raw input data (grey) and output data (black), and the output data could also be imported out-side for visualization

In PGAP-X, analytical processes are divided into three layers logically (shown as Fig. 1): data interface layer, data analysis layer, and data visualization layer. Users can easily customize parameters, manage input and output data with data interface layer. Computing was performed via the data analysis layer, and the result data would be visualized by the data visualization layer. All modules for data analysis and visualization can be organized into four parts as their functions: 1) whole genome sequences alignment and visualization of genome structure; 2) orthologous gene clustering and visualization of gene distribution by conservation; 3) pan-genome profile analysis and visualization of pan-genome profile curve; 4) genetic variants analysis and visualization from both gene and genome scale. Detailed methods and algorithms for those modules in the four parts were introduced as follows paragraphs.

### Whole genome sequences alignment and visualization of genome structure

In the data analysis module, whole genome sequences or pseudo-chromosome sequences are aligned by progressiveMauve [20]. Based on alignment result, genome structure will be visualized via data interface layer. In the visualization module, homologous DNA fragments across strains were marked with the same color. Double-clicking on the genome can align the corresponding DNA regions in other strains. Genome alignment results from other whole genome aligners are also supported for further analysis and visualization after being converted to the compatible data format.

### Orthologous gene clustering and visualization of gene distribution

In the data analysis module, a new in-house algorithm was developed to identify orthologous gene clusters among all genes across strains. The workflow for this algorithm contains three steps (shown as Additional file 1: Figure S1):

i) Parse whole genome alignment result and descending sort those homologs genomic regions by their conservation (the number of strains, who have genomic DNA sequences in this region). For those genomic regions with the same conservation, they will be sorted by the average fragment size in descending order.
ii) Cluster genes by sequence similarity and genome synteny on the same homologs genomic regions.
iii) Merge different gene clusters from step ii) by gene sequence similarities.

Both protein sequences and nucleotide sequences can be used to perform alignment, and sequence coverage and identity will be calculated based on the alignment result. Meanwhile, the threshold value for sequence coverage and identity are customizable. More detailed procedure was introduced in the Additional file 2: Supplementary document.

Orthologous gene clusters are required to visualize the gene distribution on their genomes. Each genome will be laid out horizontally, and genes are shown as colored blocks on their corresponding genomes. The color for each gene will be decided by the conservation value of the orthologous gene cluster, to which this gene was assigned. Three color models are provided in the visualization module: gradient color mode, three-color mode and four-color mode. For an analyzed bacterial population with *N* strains, the conservation value for each cluster will range from 1 to *N*. The boundaries for each color model are defined as follows:

i) Gradient color mode: *N* gradient colors will be pre-defined for those values ranging from 1 to *N*. and each gene will have one kind color based on its conservation value.
ii) Three color mode: three colors will be pre-defined, and blocks with these colors represent strain specific genes (conservation value was 1), dispensable genes (conservation value was from 2 to *N*-1) and core genes (conservation value was *N*).
iii) Four color mode: four colors will be pre-defined. Strain specific genes and core genes will use the first and the fourth colors, respectively. Dispensable genes with conservation value from 2 to $$ \raisebox{1ex}{$N$}\!\left/ \!\raisebox{-1ex}{$2$}\right. $$ share the second color and dispensable genes with conservation value from $$ \left(\raisebox{1ex}{$N$}\!\left/ \!\raisebox{-1ex}{$2$}\right.+1\right) $$ to *N*-1 share the third one.

### Pan-genome profile analysis and visualization of pan-genome profile curve

In the data analysis module, pan-genome profile will be calculated based on orthologous gene clusters from all strains, and computational methods were the same as those in PGAP. In the visualization module, the curves for pan-genome size and core gene size will be viewed in the same window. A graphic interactive interface was provided to adjust the graph.

### Genetic variants analysis and visualization from both gene and genome scale

Genetic variants in bacterial genomes will be analyzed on both genome scale and gene scale. In the data analysis module, two key parameters, substitution frequency (*f*) and substitution number (*m*) in 1 kb regions, are employed to filter out genomic regions or genic region with high substitutions. These genomic region or genic region would be displayed on the genome structure in the genome scale or gene scale models respectively.

Genetic variants analysis among pairwise or multiple genomes are also included on genome scale. For pairwise variation analysis, a reference genome should be selected first, and the remaining genomes will be taken as query genomes. All variation sites will be detected based on the whole genome alignment result. Regions with the following criterions will be outputted and displayed as high substitution regions: 1) no less than *m* substitution sites in the region, 2) substitution frequency in the region is no less than *f*, 3) the interval between any two substitution sites is no more than 1/ *f*.

For variation analysis on gene scale, MUSCLE program is utilized to align those protein sequences for all genes from the same cluster [21], and then protein sequences alignment results will be back-translated to nucleotide sequences alignment results. Variation analysis on gene scale is then performed based on the nucleotide sequences alignment result. Genes with substitution frequency no less than *f* and substitution sites no less than *m* are taken as high substitution genes. All high substitution genes will be visualized by their coordinates on the genomes.

### Test bacterial genomic datasets

The accession number for the genomic data from 14 *Streptococcus pneumonia* strains were NC_003028, NC_003098, NC_008533, NC_010380, NC_010582, NC_011072, NC_011900, NC_012466, NC_012467, NC_012468, NC_012469, NC_014251, NC_014494 and NC_014498. The accession number for the genomic data from 14 *Chlamydia trachomatis* strains were NC_007429, NC_010287, NC_017430, NC_017431, NC_017436, NC_017437, NC_020511, NC_020977, NC_021050, NC_021888, NC_021892, NC_021898, NC_022548, NC_023060. All these genomic data were downloaded from NCBI FTP.

## Result

### Implementation of PGAP-X

**Fig. 2:**
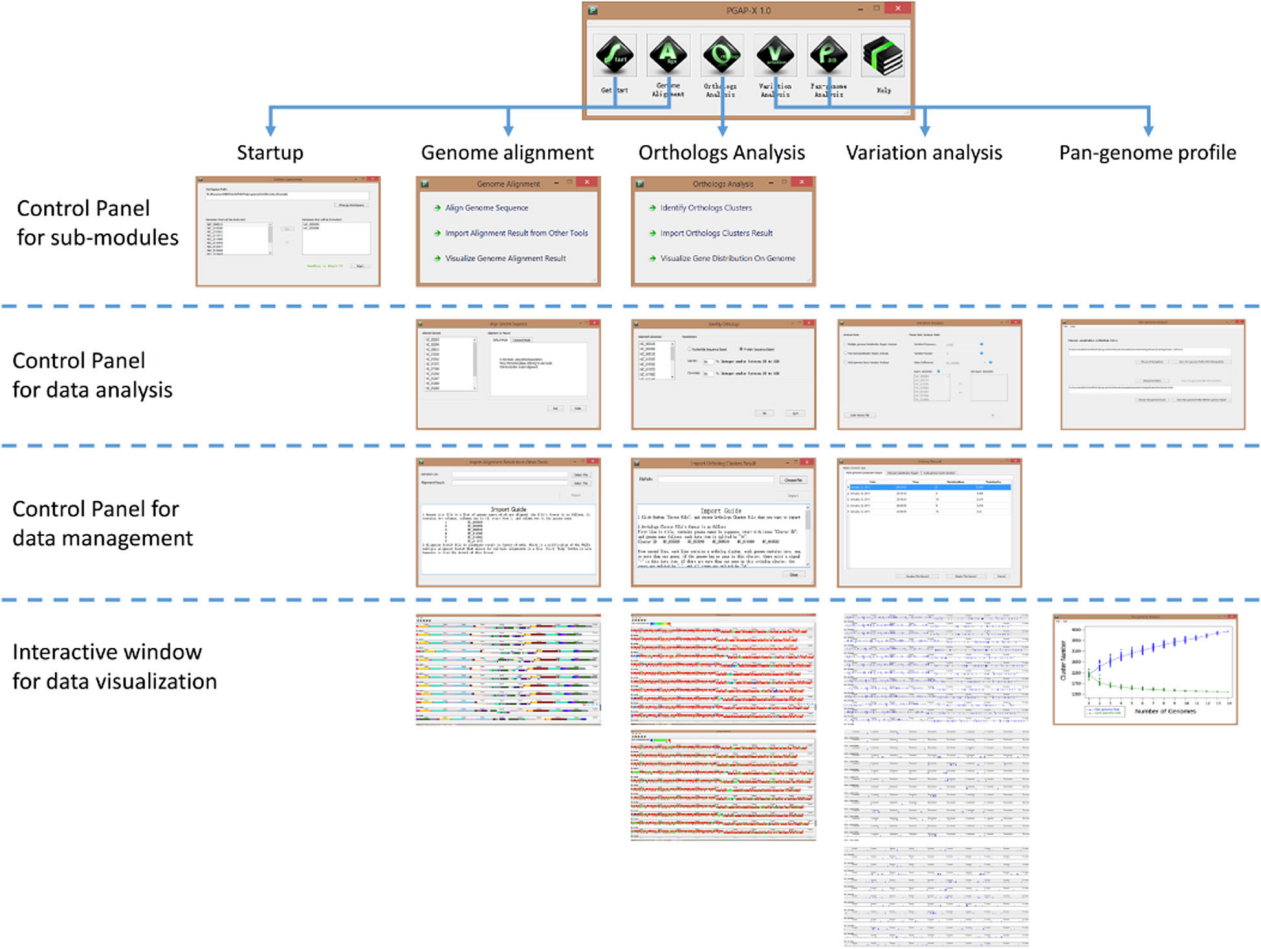
The snapshot of PGAP-X graphic interface. The interface of PGAP-X in different analytical modules and hierarchical interactive interface

Based on C++/Qt, we developed a microbial comparative genomic analysis platform with a user-friendly graphic interface, which could be run on Windows, Linux and Mac OSX platform. The snapshot for the graphic user interface, and example results are shown as Fig. 2. Based on these three logical layers, four kinds of data analysis and visualization modules have been developed from pan-genome sight. Before launching the PGAP-X program, genome sequence and gene annotation files for each strain are required as the primary input data. Whole genome alignment result and orthologous gene clusters are important secondary data in PGAP-X. Therefore, these two kinds of secondary data generated by other programs with similar functions can also be incorporated if the data is converted into a compatible format in PGAP-X.

### Performance of PGAP-X

To test the performance of PGAP-X, we downloaded genomic data of 14 *Streptococcus pneumonia* strains and 14 *Chlamydia trachomatis* strains from NCBI FTP. With the default mode and parameters, we comprehensively analyzed *S. pneumonia* strains and *C. trachomatis* strains genome sequences with PGAP-X. The analysis results present the genome diversity of these two kinds of bacteria from several sights, including genome structure, orthologous gene clusters, pan-genome profile, gene distribution, and genetic variation from both gene and genome scales.

#### Whole genome alignment and visualization of genome structure

**Fig. 3:**
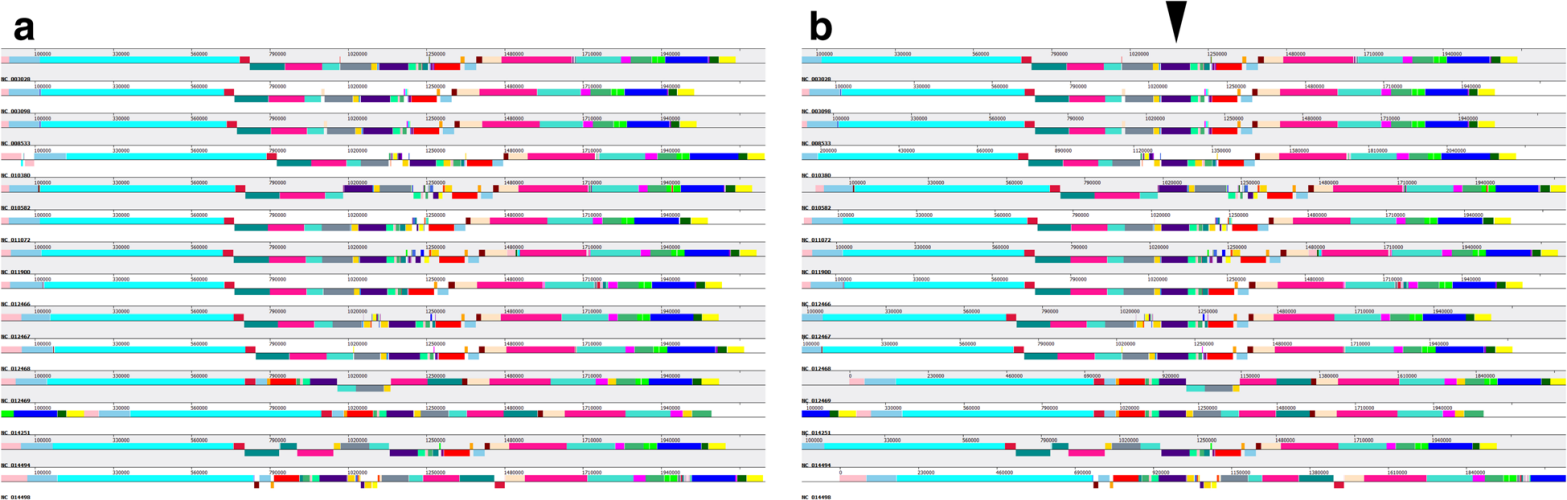
The whole genome alignment result among 14 *Streptococcus pneumoniae* strains. **a** Is the default status of the visualized alignment result, and (**b**) is the re-plotted and visualized alignment result after clicking the black triangle, and the whole genome was realigned based on the center of the clicked sites. Colored blocks are different homologous genomic fragment regions in each strain, and all DNA genomic fragments are marked with the same color with the corresponding homologous genomic fragment regions in other strains. Blocks below the center line indicate that regions are aligned in the reverse complement orientation (inversion)

Firstly, whole genome sequences alignment was performed among 14 *S. pneumonia* strains with the default mode, and results show that genomic fragment inversion events frequently occurred among genome regions range from 640 kb to 1.4 Mb (shown as Fig. 3a), which is consistent with previous report on *S. pneumonia* genomes [22]. Compared with *S. pneumonia* strains, genome structure of *C. trachomatis* strains is extremely conserved (Additional file 3: Figure S2A). Meanwhile, dynamics in genome structure can be captured by clicking different genomic fragment regions on PGAP-X graphic interface. Corresponding homology regions in different strains could be aligned on the clicked site (Fig. 3b, and Additional file 3: Figure S2B).

#### Orthologous gene clustering and gene distribution by their conservation level

Based on protein sequences, orthologous gene identification was performed among *S. pneumonia* and *C. trachomatis* respectively. The default parameters were used, which is a minimum of 50% coverage and 50% identity on the protein sequences. To evaluate the performance of the algorithm in identification of orthologous genes, PGAP was also used to detect the orthologous cluster in both *S. pneumonia* and *C. trachomatis*. Comparison of orthologous clusters from PGAP-X and PGAP was shown as Additional file 4: Table S1, and it was found that there is extremely high overlap in the orthologous clusters from these two programs. In the PGAP, both MP (using the option --method MP to clustering orthologous genes) and GF (using the option --method GF to clustering orthologous genes) methods are based on sequence similarity, which usually involving in the issue of paralogous genes. Different from programs based on sequence similarity and phylogeny [23], PGAP-X uses both genome synteny from whole genome alignment result and sequences similarity in protein sequence level to identify orthologous gene (Additional file 5: Figure S8). Therefore, paralogous genes are further distinguished by their relative locations on genome in PGAP-X (Additional file 6: Figure S3 and Additional file 7: Figure S4).

**Fig. 4:**
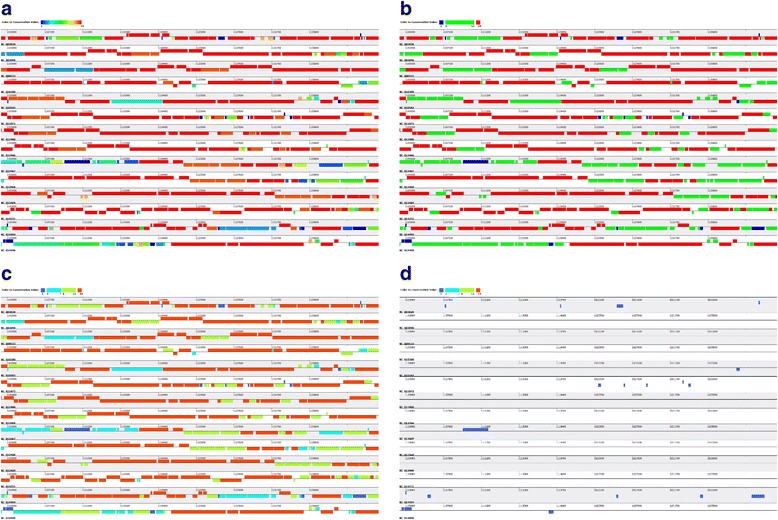
Snapshot of gene distribution by their conservation in 14 *S. pneumonia* strains genomes. Gene distribution can be presented in three kinds of models: (**a**) According to the gene conservation level, the location of all genes were filled with gradient colors; (**b**) From pan-genome sight, all genes are classified into core genes, dispensable genes, and strains specific genes, and the locations of those three classes of genes are filled with three different colors; (**c**) All genes are classified into core genes, high conserved dispensable genes, low conserved genes, and strain specific genes, and the locations of those four classes genes are filled with four different colors. **d** Is the distribution of those strain specific genes

To further analyze the diversity of gene content from their location on the genome, all genes in *S. pneumonia* strains and *C. trachomatis* strains are displayed as their genomic locations (Fig. 4 and Additional file 8: Figure S5). Gradient color mode will be taken as default color mode to present gene distribution (shown in Fig. 4a, Additional file 8: Figure S5A and E). Alternatively, another two kinds of colors models are also available, i) three colors model, in which core genes, dispensable genes, and strain specific genes were represented by different colors (shown as Fig. 4B, Additional file 8: Figure S5B and F), and ii) four colors model, in which core genes, highly shared dispensable genes, lowly shared dispensable genes, and strain specific genes are represented by different colors (shown as Fig. 4C, Additional file 8: Figure S5C and G). Moreover, with filtering function in PGAP-X, we analyzed the distribution of those strain specific genes especially by setting the gene conservation to 1. From the result for *S. pneumonia* strains (shown as Additional file 8: Figure S5D), it was apparent that many strain specific genes tend to locate as a cluster on the genome. This may suggest that these genes locating in a cluster might be introduced by horizontal gene transfer (HGT) or homologous recombination with other closely and other evolutionary processes, and this deduction is also consistent in other studies about *S. pneumonia* [3, 24–26]. In contrast, a few strain specific genes are shown in the result for *C. trachomatis* strains (shown as Additional file 8: Figure S5H).

#### Pan-genome profile

The open or close feature in the pan-genome profile indicates the diversity of gene content in the bacterial population. Comparing with closed pan-genome, open pan-genome indicates higher proportion of strain-specific genes or dispensable genes in the population. If more strains were included for analysis, the pan-genome size increases all the way in the open pan-genome population, while the pan-genome size for the close pan-genome population would increase slowly or approach to a constant number.

**Fig. 5:**
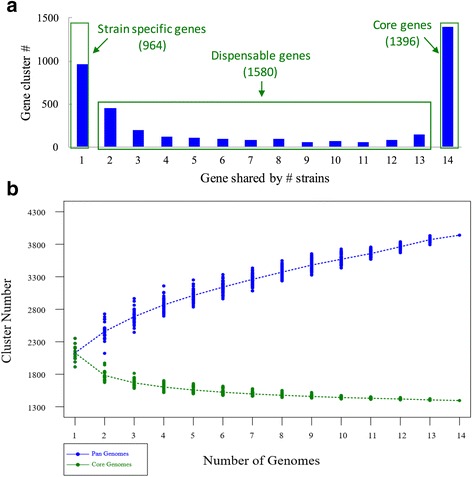
The diversity of gene contents in 14 *S. pneumonia* strains genomes. **a** Is the distribution of gene orthologous clusters with different conservation level. **b** Is the pan-genome profile curve for both pan-genome size and core genome size

Based on PGAP-X, all protein-coding genes in 14 *S. pneumonia* strains are clustered into 3940 different orthologous clusters, which are not mixed with paralogs. Among all the 3940 orthologous clusters, 35.4% (1396) are core genes clusters (those genes present in all strains), and 24.4% (964) are specific genes clusters (those genes present in only one strain) (shown as Fig. 5a). All the protein-coding genes in 14 *C. trachomatis* are clustered into 1118 clusters, with 812 (72.6%) core genes clusters and 98 (8.8%) strain specific genes clusters (shown as Additional file 9: Figure S6A). Compared to the strains with conserved genome structure, such as *Salmonella Paratyphi* A, in which 87.5% are core genes and 1.6% are strain specific genes [6], the diversity of gene content in *C. trachomatis* is still slightly higher, while it is extremely lower than S. pneumonia. To further investigate the diversity in gene content, pan-genome profile analysis was also performed by PGAP-X, and the result indicates that *S. pneumonia* owns an open pan-genome (shown as Fig. 5b), and *C. trachomatis* has relatively close pan-genome (shown as Additional file 9: Figure S6B).

#### Genetic variation on both gene and genome scale

**Fig. 6:**
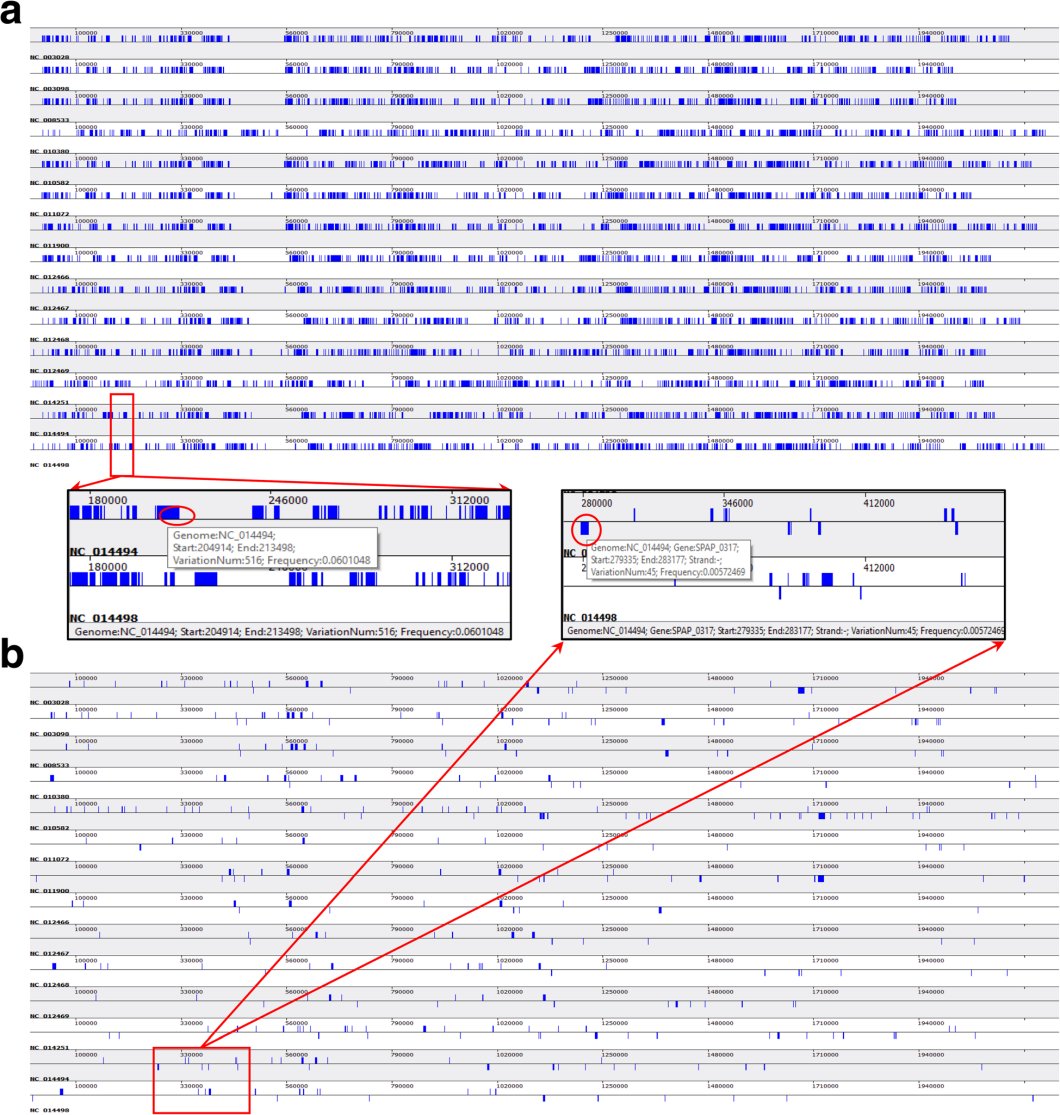
Genetic variation in 14 *S. pneumonia* strains genome from both genome and gene scale. Based on whole genome alignment, the snapshot of genetic variation on the genome scale are shown as (**a**) and the extended panel is the zoomed-in snapshot in the red rectangle. Based alignment result in each orthologous cluster, the snapshot of genetic variation on the gene scale are shown as (**b**), and the extended panel is the zoomed-in snapshot in the red rectangle. Information on the status bar is the genetic variation information on the selected region (in red circle) by right clicking

Based on whole genome alignment result and orthologous gene clusters, genetic variation analysis was performed on both genome scale and gene scale for *S. pneumonia* and *C. trachomatis*. The genomic regions and genes with high substitution rate are presented for *S. pneumonia* (Fig. 6a and b) and *C. trachomatis* (Additional file 10: Figure S7A and B) respectively. Detailed information for genetic variation on each DNA fragment or protein-coding gene can be viewed by right-clicking on the block of genomic region or gene (shown as zoomed-in snapshot in Fig. 6 and Additional file 10: Figure S7).

## Discussion

Since pan-genome concept employed in the population study of bacterial species more than 10 years ago, many different pan-genome analytical tools and databases were developed. However, a never-ending issue is how to interpret and present these result data. GView server provides a graphic interface to present core and accessory genome, including coding region information, GC content and sequence similarity [27]. However, GView does not have modules to present pan-genome profile, orthologous relationship on genome structure and genetic variation among bacterial population. As a web server, panX provides an interactive interface to visualize various data, including pan-genome statistical charts, gene cluster table, alignment, comparative phylogenies, metadata table [28]. However, it does not provide information about genome structure, gene distribution on the genome, and genetic variation. PanWeb serves as a web-based PGAP, and output results from PGAP will be presented as images by R scripts in panWeb [29]. PGAP did not integrate and interpret genome structure and gene distribution, which were also not included in panWeb consequently. To address this issue, we developed PGAP-X based on previously published software PGAP. PGAP was designed as an automatic data analysis pipeline, while PGAP-X does not only develop and integrate multiple modules for data analysis, but also modules for visualization. Moreover, analytical result data generated by other programs with similar functions could be also imported into PGAP-X to be further analyzed and visualized. In PGAP, all analyses are gene-oriented, while PGAP-X is genome-oriented. Based on whole genome alignment result, PGAP-X can exhibit bacterial diversity in genome structure, gene content, and genetic variation from pan-genome sight. In the pan-genomic analysis, orthologous cluster identification is a very essential step, as paralogs are inevitable issue in many programs. To solve this problem, a novel algorithm for identifying orthologous cluster was also developed and integrated in PGAP-X, with paralogs distinguished by the genomic location from whole genome alignment. So far, PGAP-X only supports those strains with completed genomes or pseudo-genomes (well organized and ordered super-contigs or scaffolds), while both completed genomes and draft genomes are still well supported in PGAP. In future version, we plan to develop new algorithm to align and analyze DNA fragment (contig sequences), and integrate more visualization methods for presenting the relationship between genomic structure and gene feature.

## Conclusions

Based on C++/Qt, we have implemented a cross-platform software PGAP-X with graphic interface. Multiple modules have been developed to analyze and visualize dynamics in genome structure and gene content, which will better help biologists to understand dynamics and evolution in bacterial genomes.

## Additional files

Additional file 1: Figure S1. The flowchart for the strategy of orthologs identification. (DOCX 365 kb)

Additional file 2: Supplementary document. (DOCX 22 kb)

Additional file 3: Figure S2. The whole genome alignment result among 14 C. trachomatis strains. (DOCX 809 kb)

Additional file 4: Table S1. Comparison of identical orthologous clusters from PGAP-X and PGAP. (DOCX 12 kb)

Additional file 5: Figure S8. The algorithm details for orthologous gene clustering. (DOCX 567 kb)

Additional file 6: Figure S3. Percentage of orthologous clusters with paralogs among orthologous clusters from PGAP-X and PAGP (MP and GF). (DOCX 198 kb)

Additional file 7: Figure S4. Example for distinguishing paralogs by their location on the genome. (DOCX 1836 kb)

Additional file 8: Figure S5. The location distribution of all genes by their conservation in 14 S. pneumonia strains genomes and 14 C. trachomatis strains genomes. (DOCX 6111 kb)

Additional file 9: Figure S6 The diversity of gene contents in 14 C. trachomatis strains genomes. (DOCX 793 kb)

Additional file 10: Figure S7. Genetic variation in 14 C. trachomatis strains genome from both genome and gene scale. (DOCX 623 kb)

## Abbreviations

GF: Using the option --method GF to clustering orthologous genes in PGAP
HGT: Horizontal gene transfer
MP: Using the option --method MP to clustering orthologous genes in PGAP
PGAP: Pan-genome analysis pipeline

## References

[1] Medini D (2005). The microbial pan-genome. Curr Opin Genet Dev.

[2] Tettelin H (2005). Genome analysis of multiple pathogenic isolates of *Streptococcus agalactiae*: implications for the microbial “pan-genome”. Proc Natl Acad Sci U S A.

[3] Donati C (2010). Structure and dynamics of the pan-genome of Streptococcus Pneumoniae and closely related species. Genome Biol.

[4] Reno ML (2009). Biogeography of the Sulfolobus islandicus pan-genome. Proc Natl Acad Sci U S A.

[5] Read BA (2013). Pan genome of the phytoplankton Emiliania underpins its global distribution. Nature.

[6] Liang W (2012). Pan-genomic analysis provides insights into the genomic variation and evolution of salmonella Paratyphi a. PLoS One.

[7] Aherfi S (2013). Complete genome sequence of Cannes 8 virus, a new member of the proposed family “Marseilleviridae”. Virus Genes.

[8] Dunn B (2012). Analysis of the Saccharomyces Cerevisiae pan-genome reveals a pool of copy number variants distributed in diverse yeast strains from differing industrial environments. Genome Res.

[9] Cao J (2011). Whole-genome sequencing of multiple Arabidopsis Thaliana populations. Nat Genet.

[10] Laing C (2010). Pan-genome sequence analysis using Panseq: an online tool for the rapid analysis of core and accessory genomic regions. BMC Bioinformatics.

[11] Brittnacher MJ (2011). PGAT: a multistrain analysis resource for microbial genomes. Bioinformatics.

[12] Bayjanov JR, Siezen RJ, van Hijum SA (2010). PanCGHweb: a web tool for genotype calling in pangenome CGH data. Bioinformatics.

[13] Zhao Y (2014). PanGP: a tool for quickly analyzing bacterial pan-genome profile. Bioinformatics.

[14] Benedict MN (2014). ITEP: an integrated toolkit for exploration of microbial pan-genomes. BMC Genomics.

[15] Zhao Y (2012). PGAP: pan-genomes analysis pipeline. Bioinformatics.

[16] Choo SW (2014). Genomic reconnaissance of clinical isolates of emerging human pathogen mycobacterium abscessus reveals high evolutionary potential. Sci Rep.

[17] Milani C (2013). Comparative genomics of Bifidobacterium animalis subsp. lactis reveals a strict monophyletic bifidobacterial taxon. Appl Environ Microbiol.

[18] Song L (2013). Genetic variability of mutans streptococci revealed by wide whole-genome sequencing. BMC Genomics.

[19] Xiao J (2015). A brief review of software tools for pangenomics. Genomics Proteomics Bioinformatics.

[20] Darling AE, Mau B, Perna NT (2010). ProgressiveMauve: multiple genome alignment with gene gain, loss and rearrangement. PLoS One.

[21] Edgar RC (2004). MUSCLE: multiple sequence alignment with high accuracy and high throughput. Nucleic Acids Res.

[22] Camilli R (2011). Complete genome sequence of a serotype 11A, ST62 *Streptococcus pneumoniae* invasive isolate. BMC Microbiol.

[23] Kristensen DM (2011). Computational methods for gene Orthology inference. Brief Bioinform.

[24] Hiller NL (2010). Generation of genic diversity among *Streptococcus pneumoniae* strains via horizontal gene transfer during a chronic polyclonal pediatric infection. PLoS Pathog.

[25] Prudhomme M, Libante V, Claverys JP (2002). Homologous recombination at the border: insertion-deletions and the trapping of foreign DNA in *Streptococcus pneumoniae*. Proc Natl Acad Sci U S A.

[26] Johnston C (2013). Natural genetic transformation generates a population of merodiploids in *Streptococcus pneumoniae*. PLoS Genet.

[27] Petkau A (2010). Interactive microbial genome visualization with GView. Bioinformatics.

[28] Ding W, Baumdicker F, Neher RA. panX: pan-genome analysis and exploration. Nucleic Acids Res. 2017. https://www.ncbi.nlm.nih.gov/pubmed/29077859. 10.1093/nar/gkx977 PMC5758898 29077859

[29] Pantoja Y (2017). PanWeb: a web interface for pan-genomic analysis. PLoS One.

