# Supplementary figures and images for: PGAP-X: extension on pan-genome analysis pipeline
Source: BMC Genomics. 2018 Jan 19;19(Suppl 1):36. doi: 10.1186/s12864-017-4337-7 (PMC5780747; doi:10.1186/s12864-017-4337-7)

**Additional file 1:**

**
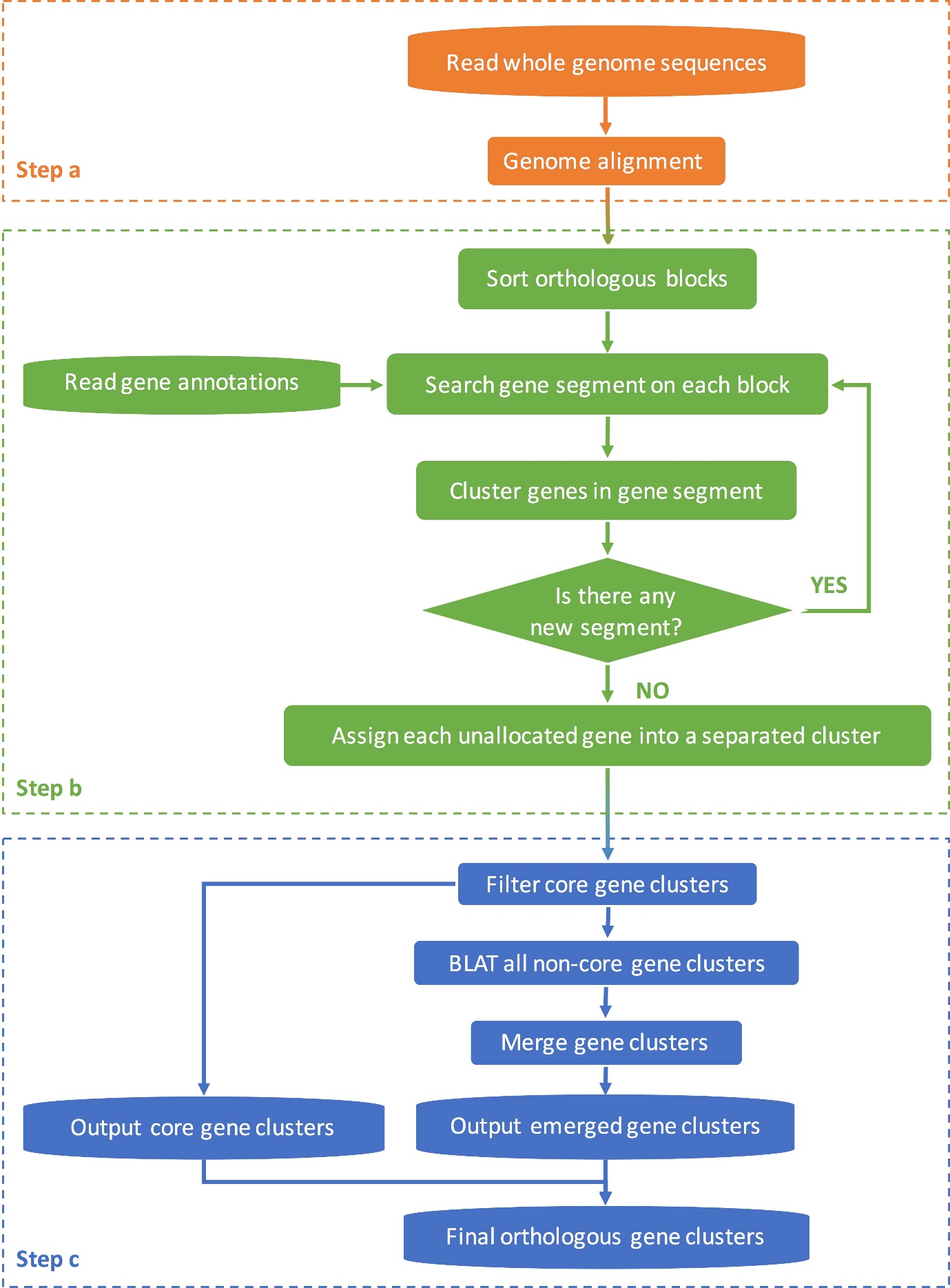
**

**Fig. S1: The flowchart for the strategy of ortholog identification.**

Supplement: Supplementary file 1 — The flowchart for the strategy of orthologs identification. (DOCX 365 kb) [file 12864_2017_4337_MOESM1_ESM.docx]
