# Supplementary material for: PGAP-X: extension on pan-genome analysis pipeline
Source: BMC Genomics. 2018 Jan 19;19(Suppl 1):36. doi: 10.1186/s12864-017-4337-7 (PMC5780747; doi:10.1186/s12864-017-4337-7)
Supplement: Supplementary file 2 — Supplementary document. (DOCX 22 kb) [file 12864_2017_4337_MOESM2_ESM.docx]

**Additional File 2: Supplementary document**

**Procedure of orthologous gene clustering**

Orthologous gene clustering in PGAP-X platform was mainly based on genome synteny and sequence similarity. The whole process includes three steps, and they are a) identifying orthologous genomic region, b) clustering genes by genome synteny and sequence similarity, c) re-clustering gene clusters based on sequence similarity. Users could select protein sequences or nucleotide sequences as gene sequences to perform alignment. Meanwhile, parameters of sequence coverage and identity were also customizable. The overview for orthologous gene clustering strategy is shown as **Additional file 1: Fig. S1**, and the details are elaborated as follows.

**Step a:** Identification of orthologous genomic region was performed by aligning all whole genome sequences of ***N*** strains (Supposing the total genome number was ***N***). Each block in the result had orthologous genomic regions from one or more strains, and each strain had at most one genomic fragment in one block.

**Step b:** Genes were clustered according to their locations on orthologous genomic region. The algorithm workflow is as follows:

1. All orthologous genomic regions (defined as blocks) were sorted in descending order by their conservation value (the number of strains, who have genomic DNA sequences in this region). If two or more blocks had the same conservation value, these blocks would be sorted in descending order by the average length of genomic DNA sequences from all strains in block.
2. Two gene pools were created, and they are named unallocated gene pool and allocated gene pool. In the beginning, all genes from *N* strains were put into the unallocated gene pool. During the clustering process, if a gene was assigned into a gene cluster, it would be moved from the unallocated gene pool to the allocated gene pool.
3. For each descending sorted block, all gene segments would be searched and calculated by scanning gene annotation information on each position at the aligned block. For each strain, function *original* (*i*) was defined as the coordination on the original whole genome sequence for the *i^th^* position on the aligned block. If no gene was annotated at the position *original* (*k*-1) and *original* (*k*+*n*+1) in all strains in the block, and one or more genes were annotated at all position from *original* (*k*-1) to *original* (*k*+*n*+1) in any strain in the blocks, then the region from position *k* to *k*+*n* on the aligned block would be taken as a gene segment. Genes were annotated at any position from *original* (*k*-1) to *original* (*k*+*n*+1) in any strain would be named as segment gene list.
4. For all the genes in the segment gene list, if any of them have been assigned into a gene cluster, this gene was removed from current the segment gene list. The remaining gene(s) in this list would be judged whether all these genes are annotated in the same strain. If so, there would be no further operation with this gene segment and segment gene list, and go to process the next gene segment. If these genes are annotated in two or more strains, go on deal with these genes with step b5).
5. For all the remaining genes, pair-wise align them, and calculate the sequence identity and coverage based on the alignment result. If the given thresholds of coverage and identity were satisfied, these two genes would be taken as homologous gene pairs. For all homologous gene pairs, Union-Find algorithm was employed to cluster them into different orthologous gene clusters, which was introduced in **Additional file 5: Fig. S8A**. In short, if gene *a* and gene *b* was homologous gene pair, gene *a* and gene *c* was homologous gene pair, then *a*, *b*, and *c* were homologous genes. If any orthologous gene cluster contained paralogs genes (*a* and *a’*), then location overlap on the block between the paralogs genes and genes in other strains would be calculated, and paralogs genes would be clustered into different gene clusters (**Additional file 5: Fig. S8B**). For all orthologous gene clusters from this gene segment, if the cluster contained one gene, this cluster was discard. And all remaining clusters were pushed into raw cluster list, and genes in the raw cluster list were moved from the unallocated gene pool to the allocated gene pool.
6. Repeat step b3), b4), b5), until no any new gene segment was found in any blocks.
7. Each gene in the unallocated gene pool was assigned as a separated gene cluster. So far, all genes were assigned into different gene clusters, and these gene clusters were taken as raw gene clusters.

**Step c:** All potential homologous gene clusters were emerged based on their sequence similarity.

1. Filter all core gene clusters (the gene number is equal to the total strains number), in which each strain had one and only one gene, and all core gene clusters directly would be taken as final orthologous clusters.
2. Sort all non-core gene clusters by their conservation value (gene number in this cluster), if two or more gene clusters have the same conservation value, sort them in descending order by average length of all genes in the cluster.
3. Align each gene in non-core gene clusters to other genes in all other non-core clusters. For example, given two clusters, A and B, include $n_{A}$and $n_{B}$ genes respectively. If there were no less than $\frac{n_{A}}{2}$ genes in cluster A were homologous gene pair with genes in cluster B, and there were also no less than $\frac{n_{B}}{2}$ genes in cluster B were homologous gene pair with genes in cluster A, cluster A and cluster B were potential homologous clusters. The average matched nucleotide (or amino acid) number in all homologous gene pairs for genes between cluster A and cluster B would be defined as the cluster match value between cluster A and cluster B.
4. Use the first cluster in the descending sorted non-core clusters as seed cluster; extract all potential homologous clusters with the seed cluster and make them as a candidate list; descending sort the candidate list by the cluster match value between them and the seed cluster; pick out the first gene cluster (named test cluster) from the sorted candidate list, if there was a strain, which had gene in test cluster and seed cluster simultaneously, remove this cluster from the candidate list, otherwise emerge seed cluster and test cluster to a new seed cluster; check whether the next cluster in the candidate list should be emerged with seed cluster until the candidate list was empty; output the final emerged cluster as orthologous cluster.
5. Repeat the process in **c4**), until all non-core clusters were analyzed.
