# Supplementary material for: PGAP-X: extension on pan-genome analysis pipeline
Source: BMC Genomics. 2018 Jan 19;19(Suppl 1):36. doi: 10.1186/s12864-017-4337-7 (PMC5780747; doi:10.1186/s12864-017-4337-7)
Supplement: Supplementary file 3 — The whole genome alignment result among 14 C. trachomatis strains. (DOCX 809 kb) [file 12864_2017_4337_MOESM3_ESM.docx]

**Additional file 3:**


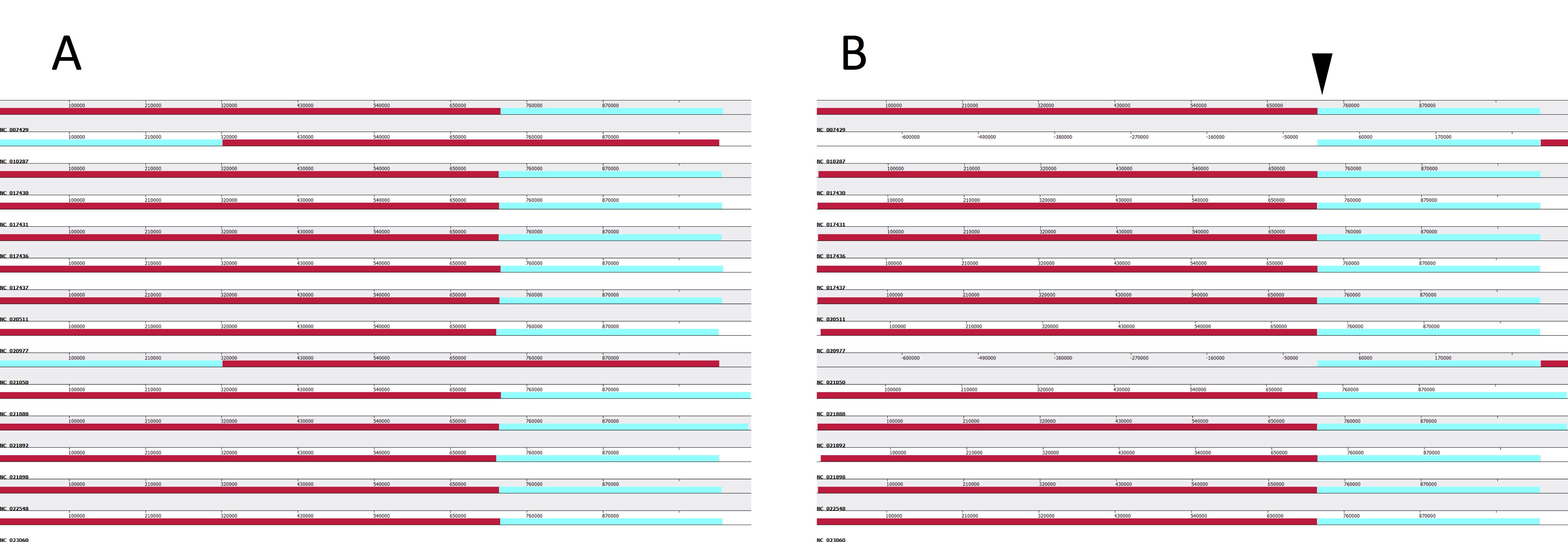


**Fig. S2: The whole genome alignment result among 14 C. trachomatis strains.**

(A) is the default alignment result data, and (B) is the re-aligned results based on the center of the clicked sites. Colored blocks are different homologous genomic fragment regions in each strain, and all DNA genomic fragments are marked with the same color with the corresponding homologous genomic fragment regions in other strains. Blocks below the center line indicate that regions are aligned in the reverse complement orientation (inversion).
