# Supplementary material for: PGAP-X: extension on pan-genome analysis pipeline
Source: BMC Genomics. 2018 Jan 19;19(Suppl 1):36. doi: 10.1186/s12864-017-4337-7 (PMC5780747; doi:10.1186/s12864-017-4337-7)
Supplement: Supplementary file 4 — Comparison of identical orthologous clusters from PGAP-X and PGAP. (DOCX 12 kb) [file 12864_2017_4337_MOESM4_ESM.docx]

**Additional File 4**

Table S1: Comparison of identical orthologous clusters from PGAP-X and PGAP

|  | GF or MP from PGAP | PGAP-X | Species |
| --- | --- | --- | --- |
| GF@PGAP vs PGAP-X | 93.2% | 95.4% | *C. trachomatis* |
| MP@PGAP vs PGAP-X | 97.8% | 96.8% |  |
|  |  |  |  |
| GF@PGAP vs PGAP-X | 90.7% | 80.8% | *S. pneumoniae* |
| MP@PGAP vs PGAP-X | 88.6% | 80.5% |  |
