# Supplementary material for: PGAP-X: extension on pan-genome analysis pipeline
Source: BMC Genomics. 2018 Jan 19;19(Suppl 1):36. doi: 10.1186/s12864-017-4337-7 (PMC5780747; doi:10.1186/s12864-017-4337-7)
Supplement: Supplementary file 5 — The algorithm details for orthologous gene clustering. (DOCX 567 kb) [file 12864_2017_4337_MOESM5_ESM.docx]

**Additional file 5:**


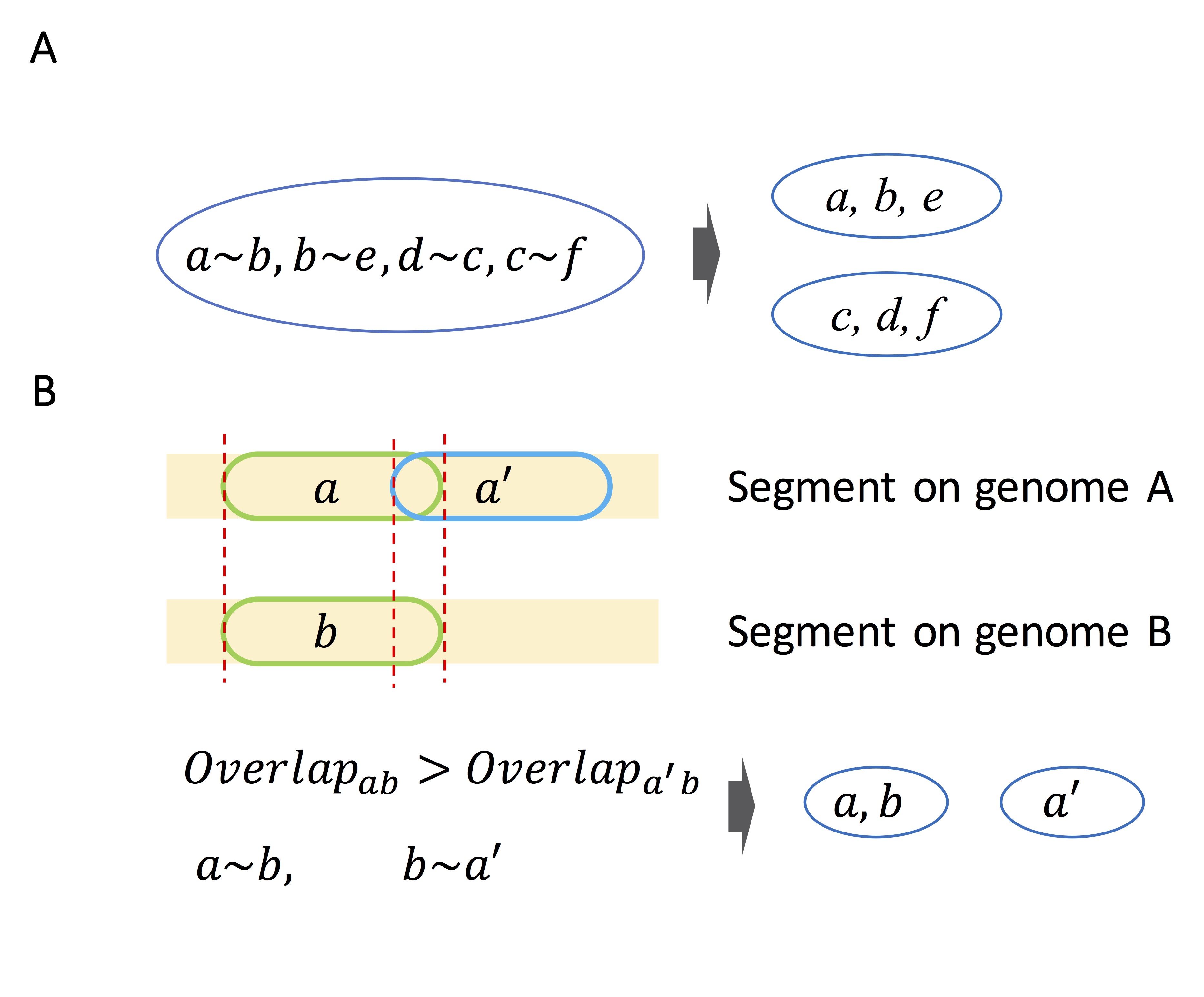


**Fig. S8: The algorithm details for orthologous gene clustering.**

a, a’ b, c, d, e, f represents genes, and the sign “~” indicates that the alignment result (aligned coverage and identity) of the two genes satisfies the threshold. (A) Based on the pairwise similarity values, cluster genes into different gene cluster with a Union-Find like algorithm. (B) Discriminate paralogs in the same segment based on the length of overlap region on aligned genome sequence.
