# Supplementary material for: PGAP-X: extension on pan-genome analysis pipeline
Source: BMC Genomics. 2018 Jan 19;19(Suppl 1):36. doi: 10.1186/s12864-017-4337-7 (PMC5780747; doi:10.1186/s12864-017-4337-7)
Supplement: Supplementary file 6 — Percentage of orthologous clusters with paralogs among orthologous clusters from PGAP-X and PAGP (MP and GF). (DOCX 198 kb) [file 12864_2017_4337_MOESM6_ESM.docx]

**Additional file 6:**

**
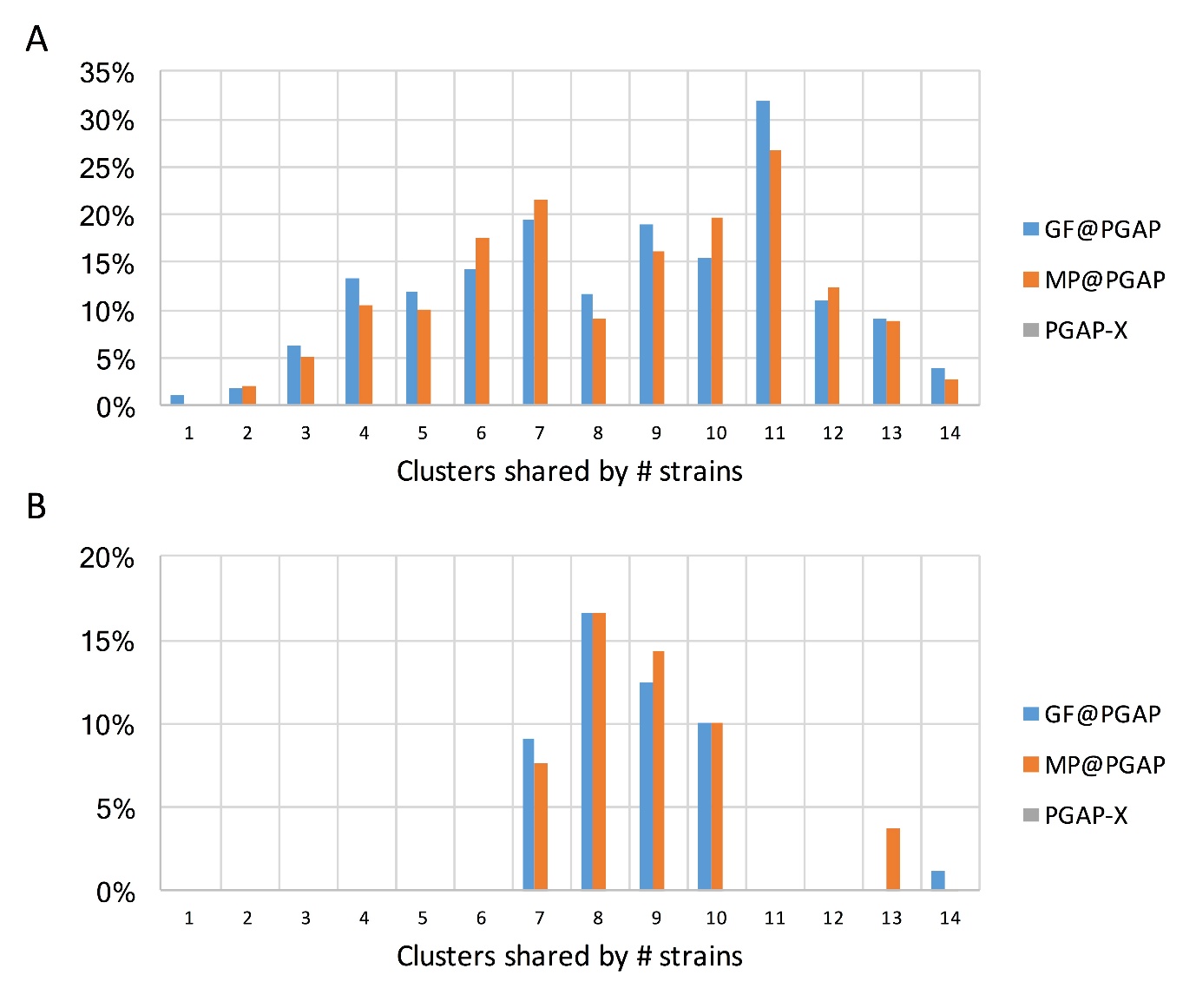
**

**Fig. S3: Percentage of orthologous clusters with paralogs among orthologous clusters from PGAP-X and PAGP (MP and GF). A** and **B** show the results from 14 *S. pneumonia* strains and 14 *C. trachomatis* strains respectively. MP (using the option --method MP to clustering orthologous genes) and GF (using the option --method GF to clustering orthologous genes) methods in PGAP are presented as GF@PGAP and MP@PGAP.
