# Supplementary material for: PGAP-X: extension on pan-genome analysis pipeline
Source: BMC Genomics. 2018 Jan 19;19(Suppl 1):36. doi: 10.1186/s12864-017-4337-7 (PMC5780747; doi:10.1186/s12864-017-4337-7)
Supplement: Supplementary file 7 — Example for distinguishing paralogs by their location on the genome. (DOCX 1836 kb) [file 12864_2017_4337_MOESM7_ESM.docx]

**Additional file 7:**


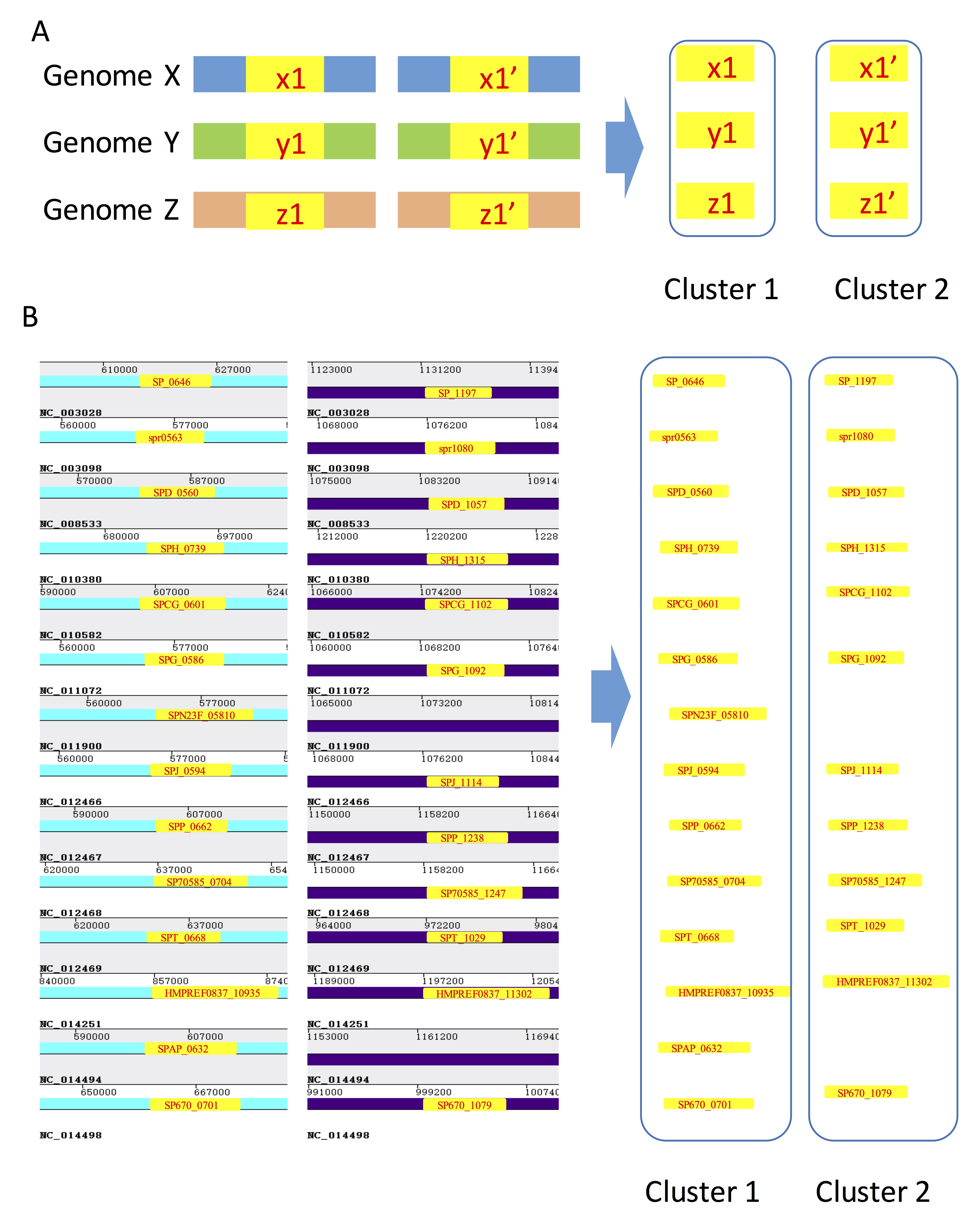


**Fig. S4: Example for distinguishing paralogs by their location on the genome.**

(A) Sketch for distinguishing paralogs by their location on the genome. X, Y and Z are different bacterial genomes. x1, x1’ are paralogs in X, y1 and y1’ are paralogs in Y, and z1 and z1’ are paralogs in Z. x1, x1’, y1, y1’, z1 and z1’ are homologous genes based on sequence similarly. x1, y1, and z1 locate on the same homologous genomic fragment regions, and x1’, y1’ and z1’ locate on another homologous genomic fragment regions. (B) A real example about how PGAP-X assigns paralogous genes into two different clusters. All these 26 genes are clustered into the same cluster in PGAP by MP and GF method.
