# Supplementary material for: PGAP-X: extension on pan-genome analysis pipeline
Source: BMC Genomics. 2018 Jan 19;19(Suppl 1):36. doi: 10.1186/s12864-017-4337-7 (PMC5780747; doi:10.1186/s12864-017-4337-7)
Supplement: Supplementary file 8 — The location distribution of all genes by their conservation in 14 S. pneumonia strains genomes and 14 C. trachomatis strains genomes. (DOCX 6111 kb) [file 12864_2017_4337_MOESM8_ESM.docx]

**Additional file 8:**


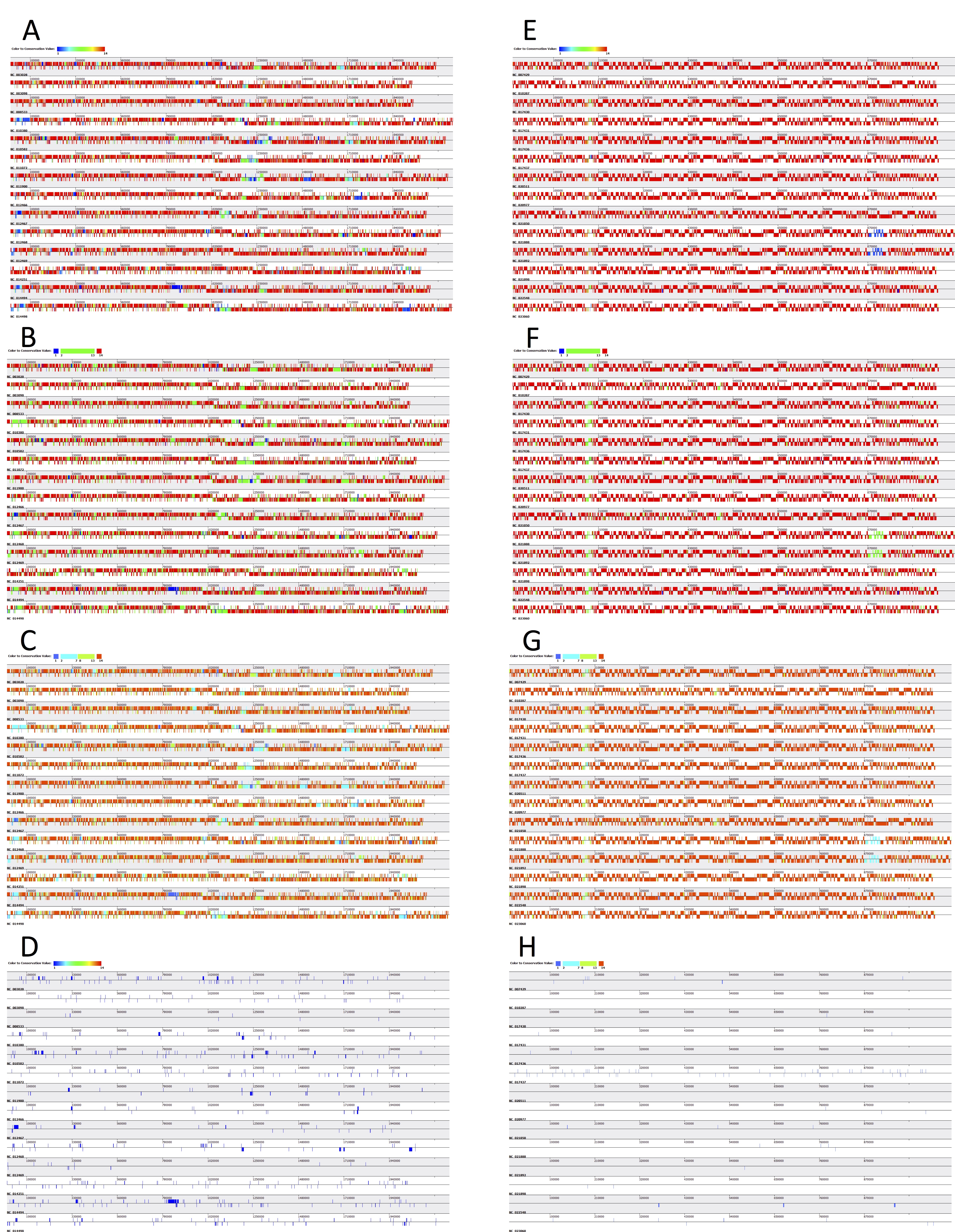


**Fig. S5: The location distribution of all genes by their conservation in 14 S. pneumonia strains genomes and 14 C. trachomatis strains genomes.**

(A), (B), (C) and (D) are the gene distribution in 14 S. pneumonia strains, and (E), (F), (G) and (H) are the gene distribution in 14 C. trachomatis. Gene distribution can be presented in three kinds of models: 1) According to the gene conservation level, the location of all genes were filled with gradient colors (A and E); 2) From pan-genome sight, all genes are classified into core genes, dispensable genes, and strains specific genes, and the locations of those three classes of genes are filled with three different colors (B and F); 3) All genes are classified into core genes, high conserved dispensable genes, low conserved genes, and strain specific genes, and the locations of those four classes genes are filled with four different colors (C and G). (D) and (H) are the distribution of those strain specific genes.
