# Supplementary material for: PGAP-X: extension on pan-genome analysis pipeline
Source: BMC Genomics. 2018 Jan 19;19(Suppl 1):36. doi: 10.1186/s12864-017-4337-7 (PMC5780747; doi:10.1186/s12864-017-4337-7)
Supplement: Supplementary file 9 — The diversity of gene contents in 14 C. trachomatis strains genomes. (DOCX 793 kb) [file 12864_2017_4337_MOESM9_ESM.docx]

**Additional file 9:**


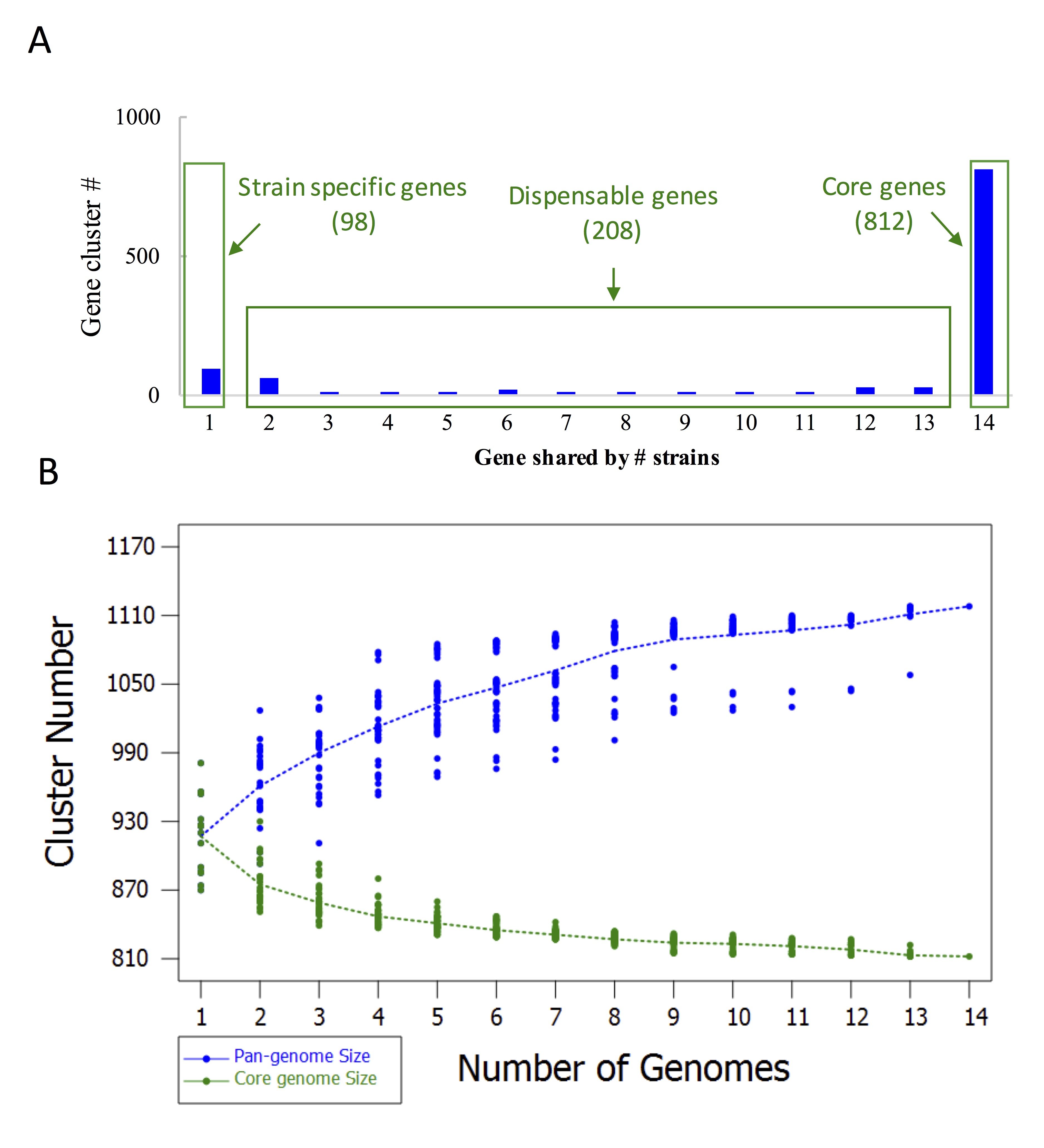


**Fig. S6: The diversity of gene contents in 14 *C. trachomatis* strains genomes.**

(**A**) is the distribution of gene orthologous clusters with different conservation level. (**B**) is the pan-genome profile curve for both pan-genome size and core genome size.
