# Supplementary material for: PGAP-X: extension on pan-genome analysis pipeline
Source: BMC Genomics. 2018 Jan 19;19(Suppl 1):36. doi: 10.1186/s12864-017-4337-7 (PMC5780747; doi:10.1186/s12864-017-4337-7)
Supplement: Supplementary file 10 — Genetic variation in 14 C. trachomatis strains genome from both genome and gene scale. (DOCX 623 kb) [file 12864_2017_4337_MOESM10_ESM.docx]

**Additional file 10:**

**
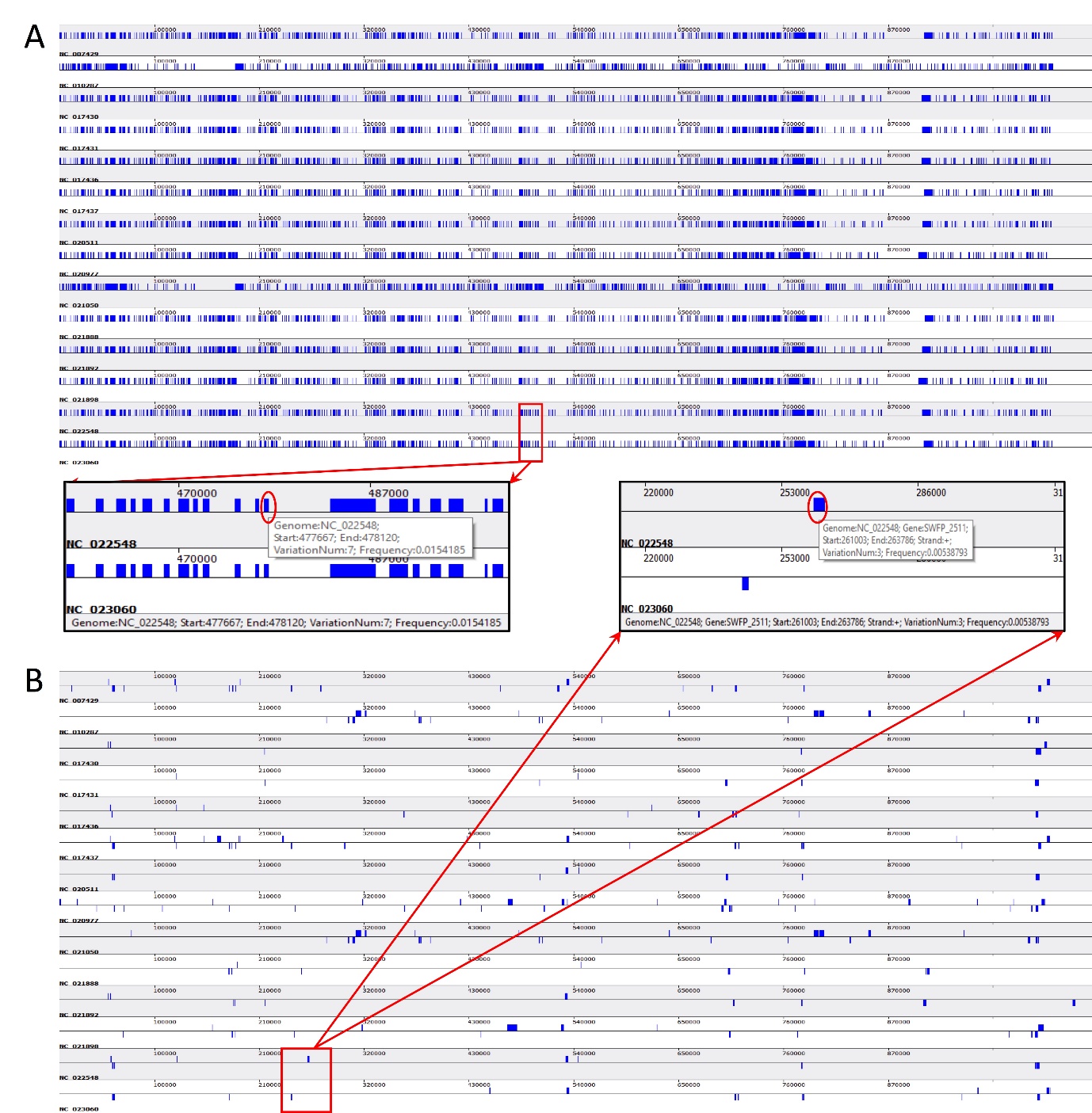
**

**Fig. S7: Genetic variation in 14 C. trachomatis strains genome from both genome and gene scale.**

Based on whole genome alignment, the snapshot of genetic variation on the genome scale are shown as (A) and the extended panel is the zoomed-in snapshot in the red rectangle. Based alignment result in each orthologous cluster, the snapshot of genetic variation on the gene scale are shown as (B), and the extended panel is the zoomed-in snapshot in the red rectangle. Information on the status bar is the genetic variation information on the selected region (in red circle) by right clicking.
